# Congophilic fibrillary glomerulonephritis recurrence post-renal transplant: diagnostic challenges and proteomic insights

**DOI:** 10.1007/s13730-025-01064-4

**Published:** 2026-01-23

**Authors:** Hiroshi Watanabe, Michiko Nagamine, Yukako Shintani-domoto, Kunio Kawanishi, Kousuke Ishino, Tsukasa Nakamura, Shinji Sumiyoshi, Eiichi Konishi

**Affiliations:** 1 Department of Surgical Pathology, Kyoto Prefectural University of Medicine, Kyoto, Japan; 2 Department of Diagnostic Pathology, Nippon Medical School Hospital, Tokyo, Japan; 3 Department of Anatomy, Showa Medical University School of Medicine, Tokyo, Japan; 4 Department of Integrated Diagnostic Pathology, Nippon Medical School, Tokyo, Japan; 5 Division of Transplant Surgery, Department of Surgery, Massachusetts General Hospital, Boston, USA; 6 Department of Organ Transplantation and General Surgery, Kyoto Prefectural University of Medicine, Kyoto, Japan; 7 Department of Diagnostic Pathology, Tenri Hospital, Nara, Japan

**Keywords:** Amyloidosis, Amyloid signature proteins, Congo red, DnaJ heat shock protein family (Hsp40) member B9 (DNAJB9), Fibrillary glomerulonephritis (FGN), LMD-LC-MS/MS, Recurrence after transplant

## Abstract

Fibrillary glomerulonephritis (FGN) is a rare kidney disease characterized by the deposition of Congo red (CR) negative fibrils measuring 12 to 24 nm in diameter on electron microscopy. Recently, DnaJ heat shock protein family (Hsp40) member B9 (DNAJB9) detected by liquid chromatography-tandem mass spectrometry (LC-MS/MS) was discovered to be a sensitive and specific biomarker for FGN. This report presents the first documented case of recurrent congophilic FGN, a rare glomerular disease with features resembling amyloidosis, occurring two years and eight months after a renal transplant. Diagnosis of both primary and recurrent FGN was confirmed through DNAJB9 detection using advanced laser microdissection and LC-MS/MS techniques. The recurrent FGN exhibited features consistent with amyloidosis, including CR positivity, characteristic microscopic findings, and fibrils on electron microscopy with a 9 to 15 nm diameter. This case demonstrates that FGN could recur in the transplanted kidney as congophilic GN with characteristic findings of amyloidosis, and it is difficult, if not impossible, to render a correct diagnosis without LC-MS/MS or immunohistochemistry.

## Introduction

Fibrillary glomerulonephritis (FGN), first reported in 1977 as a case of nephrotic syndrome [1], is a rare disease found in about 0.5 to 1% of renal biopsies [2, 3]. The patients are typically in their 50s to 60s, presenting with proteinuria, nephrotic syndrome, hematuria, hypertension, and impaired renal function [4]. Its association with malignancies, hepatitis C, autoimmune disease, and monoclonal immunoglobulinemia has been reported [5]. The prognosis of FGN is poor; half the patients become end-stage renal disease (ESRD) within four years of diagnosis [3]. Recurrent FGN after renal transplantation is exceedingly rare, with only isolated cases reported in the literature [6]. In a multi-institutional cohort study including 266 cases of confirmed and suspected FGN, there was only one case of recurrent FGN in the allograft kidney. In contrast, no recurrence was seen in 7 transplanted cases with a median follow-up time of 7 years [7].

Histologically, FGN shows a variety of glomerulonephritis, such as diffuse sclerosing, diffuse proliferative glomerulonephritis, membranoproliferative glomerulonephritis, mesangial proliferation, membranous glomerulonephritis [3]. IgG and C3 depositions in one study are positive on immunofluorescence assays (IF) in 100% and 92% of the cases, respectively [3]. Electron microscopy (EM) shows unbranched, randomly arranged fibrils with diameters of 12–24 nm in the glomerular basement membrane (GBM) and the mesangial regions [2]. Traditionally, the diagnosis of FGN has been made by Congo red (CR) negativity and identification of microfibrils on EM with larger diameters than those of amyloid fibrils, which are 8 to 10 nm in diameter.

In 2018, Dasari et al. reported DnaJ heat shock protein family (Hsp40) member B9 (DNAJB9) as a specific protein found in FGN using mass spectrometry [8]. In the same year, Nasr et al. reported that positivity in DNAJB9 immunostaining could diagnose FGN with a sensitivity of 98% and specificity of ≥ 99% [9]. Although the discovery of DNAJB9 redefined FGN as a more distinct entity, the pathogenesis of FGN is yet to be elucidated.

Here, we present a case of congophilic FGN that recurred in the allograft kidney after two years and eight months post-transplant.

## Case report

### Clinical summary

A 60-year-old male developed significant proteinuria two years and eight months after undergoing a living donor kidney transplant. His past medical history included proteinuria since he was in his teens and hypertension since he was around 20 years of age. He had no history of monoclonal gammopathy, hepatitis C virus infection, malignancy, or autoimmune diseases. He was followed up without any interventions. At the age of 40, he developed nephrotic syndrome and was treated with prednisolone for a short time. The following year, a renal biopsy was performed. This was the only biopsy performed on his native kidney. His renal function worsened, and he reached ESRD in his late 50s. He received a living kidney transplant from his family (blood type incompatible, cross-match negative). One year and eleven months after the transplant, to alleviate calcineurin inhibitor toxicity, the cyclosporine A dose was reduced, and everolimus was started at a lower dose. The graft function had remained stable: Cr 0.7-1.0 mg/dl, BUN 9.0–17.0 mg/dl, and his urinary protein level remained around 0.3 g/gCr for two years after transplantation. Four post-transplant protocol biopsies (at 1 hour, 1 month, 1, and 2 years) were performed, and no findings suspicious for recurrence of any primary or secondary disease were evident histologically. The patient developed progressive proteinuria, reaching 7.15 g/gCr within six months, with negative hematuria prompting a diagnostic kidney biopsy. Clinically, the everolimus-induced nephrotic syndrome was suspected, and everolimus was discontinued. After that, the increase in proteinuria stopped and remained between 3.5 and 6.2 g/gCr. The serum albumin level (2.7–4.8 g/dL) and eGFR (67–76) remained stable regardless of the amount of urinary protein. Two years and eight months after transplant, a kidney biopsy was performed to investigate the cause of proteinuria. The patient is in a stable condition one year after the biopsy.

### Pathological findings

1. Native Kidney
  Microscopically, mesangial proliferative glomerulonephritis was seen. CR seemed weakly positive under a light microscope but showed no apple-green birefringence under polarized light (Fig. 1A-B). The IF was positive for IgG and C3, and negative for IgA, IgM, C1q, and fibrinogen. IF for kappa and lambda light chains were not performed on the native kidney specimen (Fig. 1C-D). EM showed severe GBM thickening and microfibril deposition in the GBM and the mesangial area (Fig. 1E). The diameter of the fibrils was about 10–15 nm, which is slightly greater than that of amyloid fibrils (8–10 nm) and smaller than that of FGN (12–24 nm) (Fig. 1F). The fibrils protruded toward the epithelial side of the GBM, eroding and destroying the GBM. The diagnosis at that point was a glomerular deposition disease of unknown cause.
  **Fig. 1:**
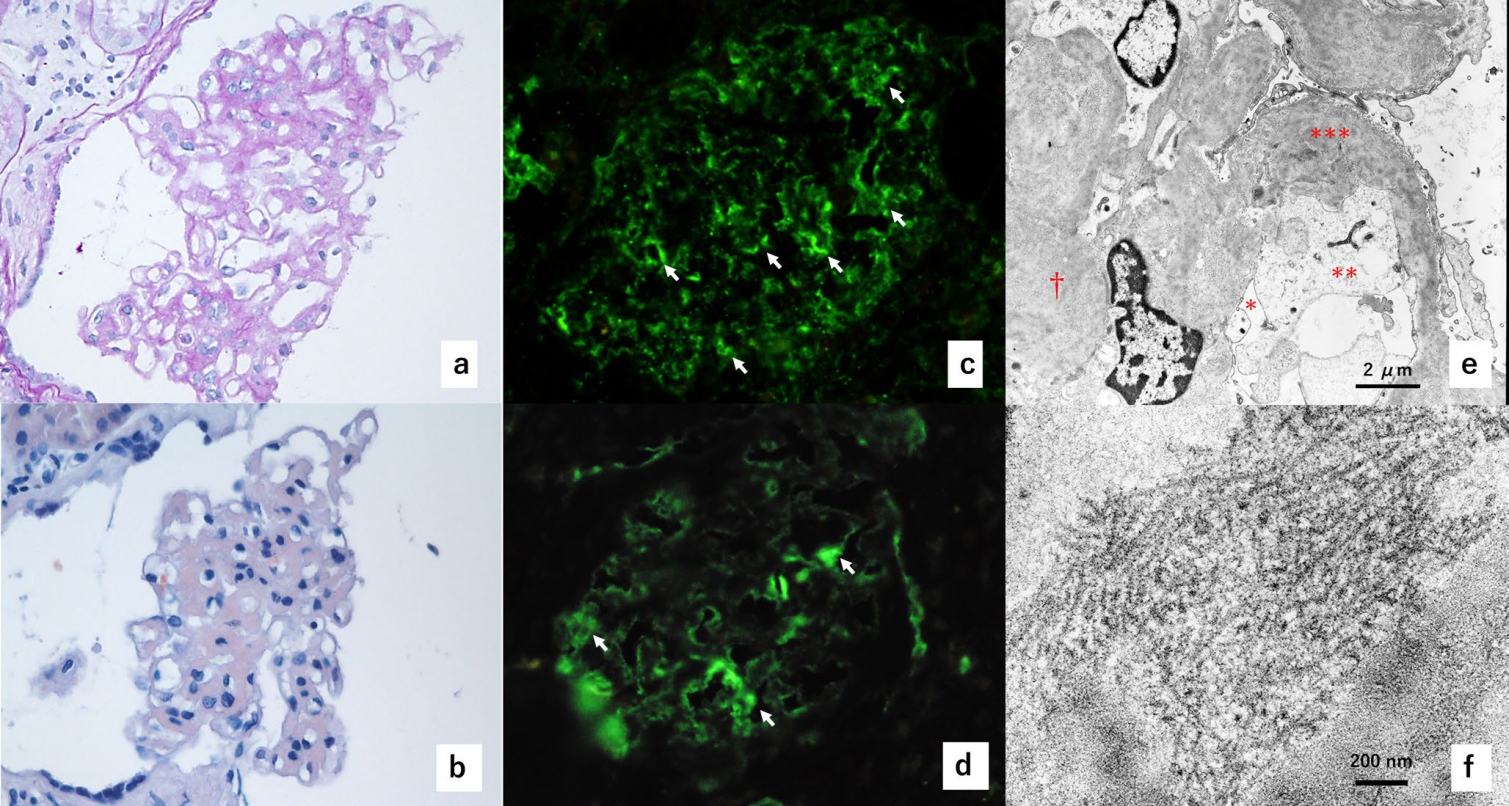
Findings on biopsy of native kidney. Original magnification, ×400 for A) and B). **A** The native kidney showed mesangial proliferation on the PAS stain. **B** The glomerular tuft showed thickening, but there was no green birefringence under polarized light on the Congo red stain. The IF was positive for **C** IgG and **D** C3. **E** EM showed severe GBM thickening and microfibril deposition in the GBM and the mesangial area. *, fenestrae; **, endothelial cells; ***, GBM; †, mesangium (×3000). **F** The diameter of the fibrils was 10–15 nm (×20000)
2. Transplanted Kidney
  On microscopic examination, there was no expansion of the mesangium, endocapillary proliferation, or GBM doubling (Fig. 2A). Periodic acid-methenamine-silver (PAM) stain showed focal/segmental spiculae that extended outward from GBM (Fig. 2B). CR was weakly positive on the mesangium and capillary wall focally and segmentally with apple-green birefringence under polarized light (Fig. 2C-D). With suspicion of amyloidosis, we performed immunohistochemical stainings for amyloid typing, but amyloid kappa, amyloid lambda, amyloid A, ATTR, and β2 microglobulin were all negative. So, laser microdissection (LMD)-liquid chromatography-tandem mass spectrometry (LC-MS/MS) was necessary for more accurate amyloid typing [10]. IgG and C3 were positive around the vascular pole and mesangium on IF (Fig. 2E-F), and negative for IgA, IgM, C1q, and fibrinogen. IF for kappa and lambda light chains were not performed on the transplanted kidney specimen. EM revealed fibrillar structures in the subepithelial, subendothelial, and mesangial regions (Fig. 3A). The width of the fibrils varied from 9 to 15 nm with a random arrangement and no branching (Fig. 3B).
  **Fig. 2:**
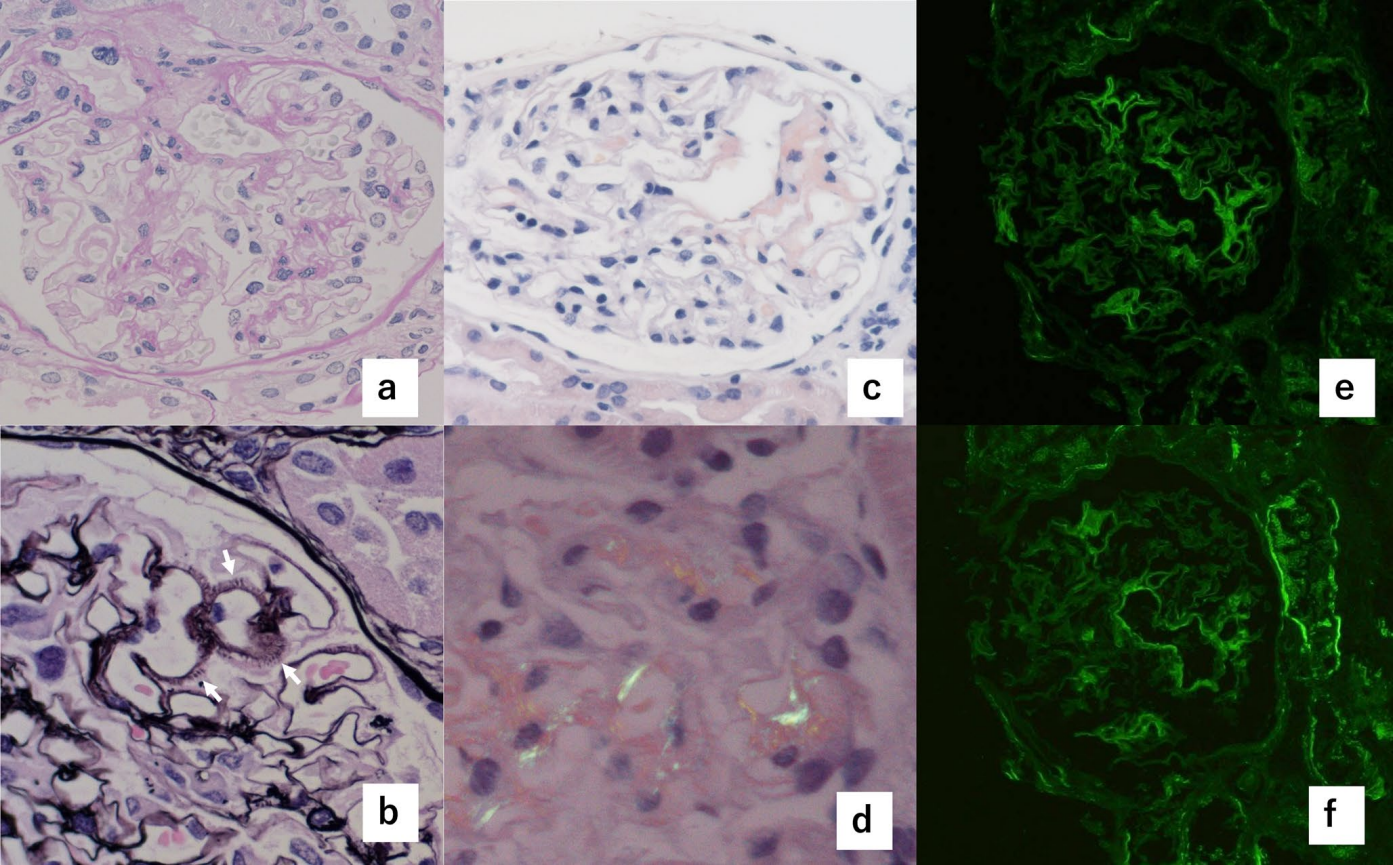
Findings on biopsy of transplanted kidney, taken two years and eight months post-transplant. Original magnification, ×400 for A) through D). **A** No aberrant proliferation of mesangial cells is seen on the PAS stain. **B** PAM stain shows focal/segmental spiculae extending outwards from the GBM. **C** Congo red was positive on mesangium and glomerular tuft. **D** Congo red stain shows green birefringence under polarized light. Immunofluorescence was positive around the vascular pole and mesangium for **E** IgG and **F** C3
  **Fig. 3:**
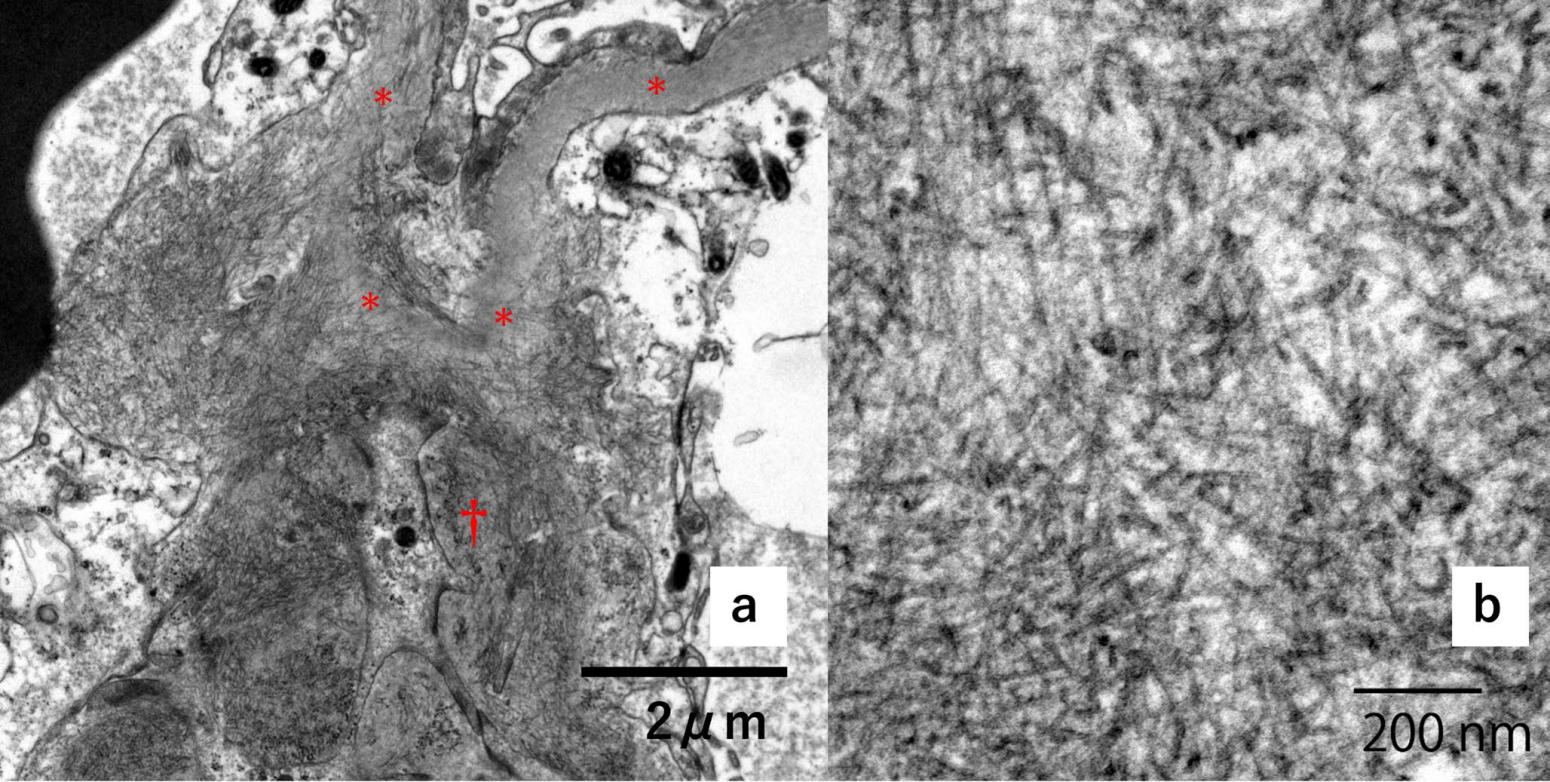
Electron microscopy revealed (**A**) fibrillar structures in the segmental subepithelial, subendothelial, and mesangial region (×8000). *, glomerular basement membrane; †, mesangium. **B** The diameter of the fibrils was 9–15 nm with a random arrangement and no branching (×50000)
  LMD and LC-MS/MS were performed, as previously described [10], in three different areas: CR-positive areas of a glomerulus (Fig. 4A, B), CR-negative areas, and tubulointerstitium. DNAJB9 was detected both in the CR-positive and CR-negative areas but not in the tubulointerstitium (Fig. 5). DNAJB9 was also detected in the native kidney (Fig. 6). Based on these findings, we diagnosed FGN in the native and transplanted kidneys.
  **Fig. 4:**
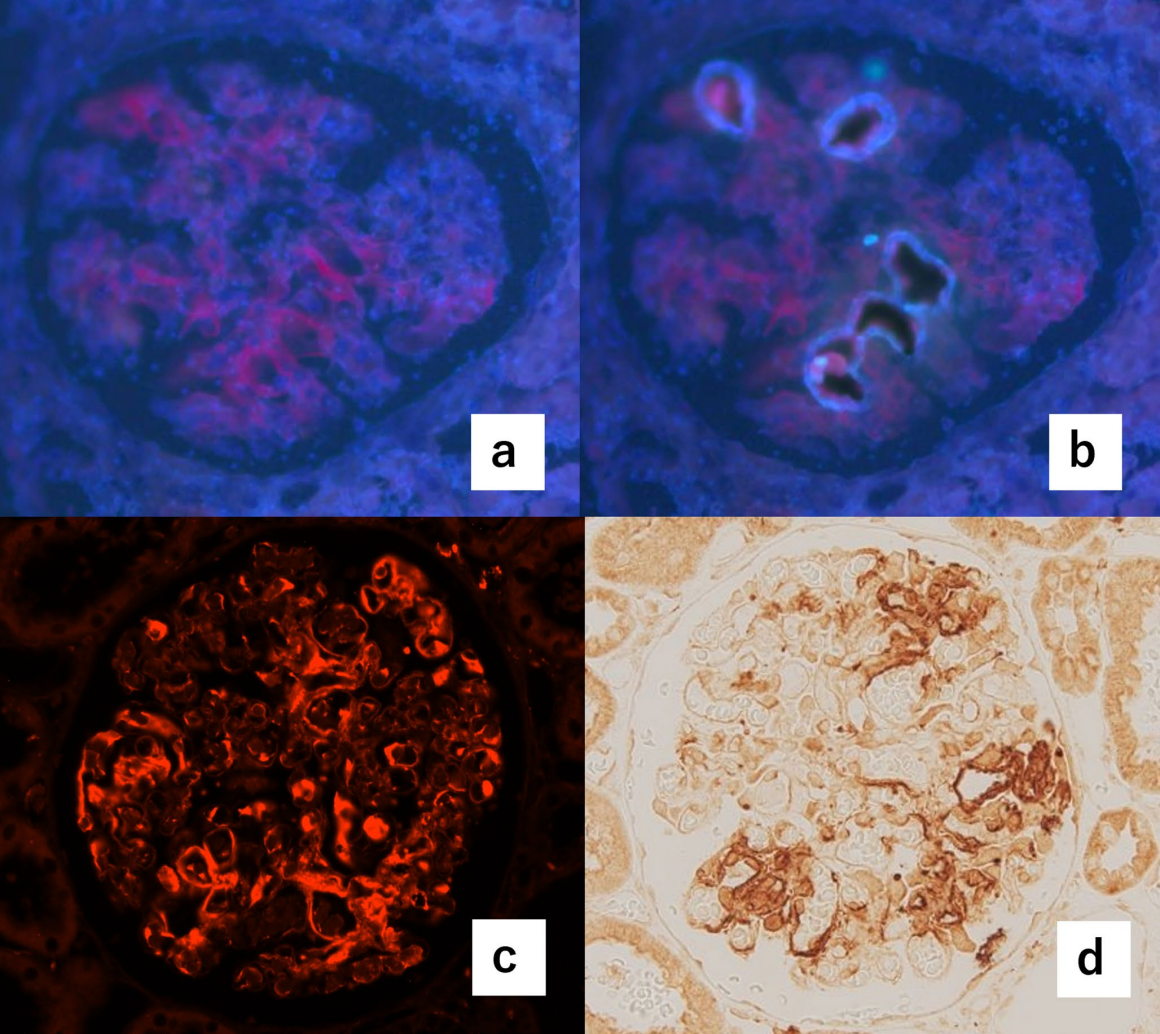
Transplanted kidney. Sampling area for MS (**A**, **B**). Immunofluorescence for SAP (**C**) and immunohistochemistry for DNAJB9 (**D**). A, B) Only areas showing Congo red positivity were collected by laser microdissection for mass spectrometry. C) SAP immunofluorescence shows positivity in the mesangial region. D) DNAJB9 immunohistochemical stain shows positivity in the mesangial region
  **Fig. 5:**
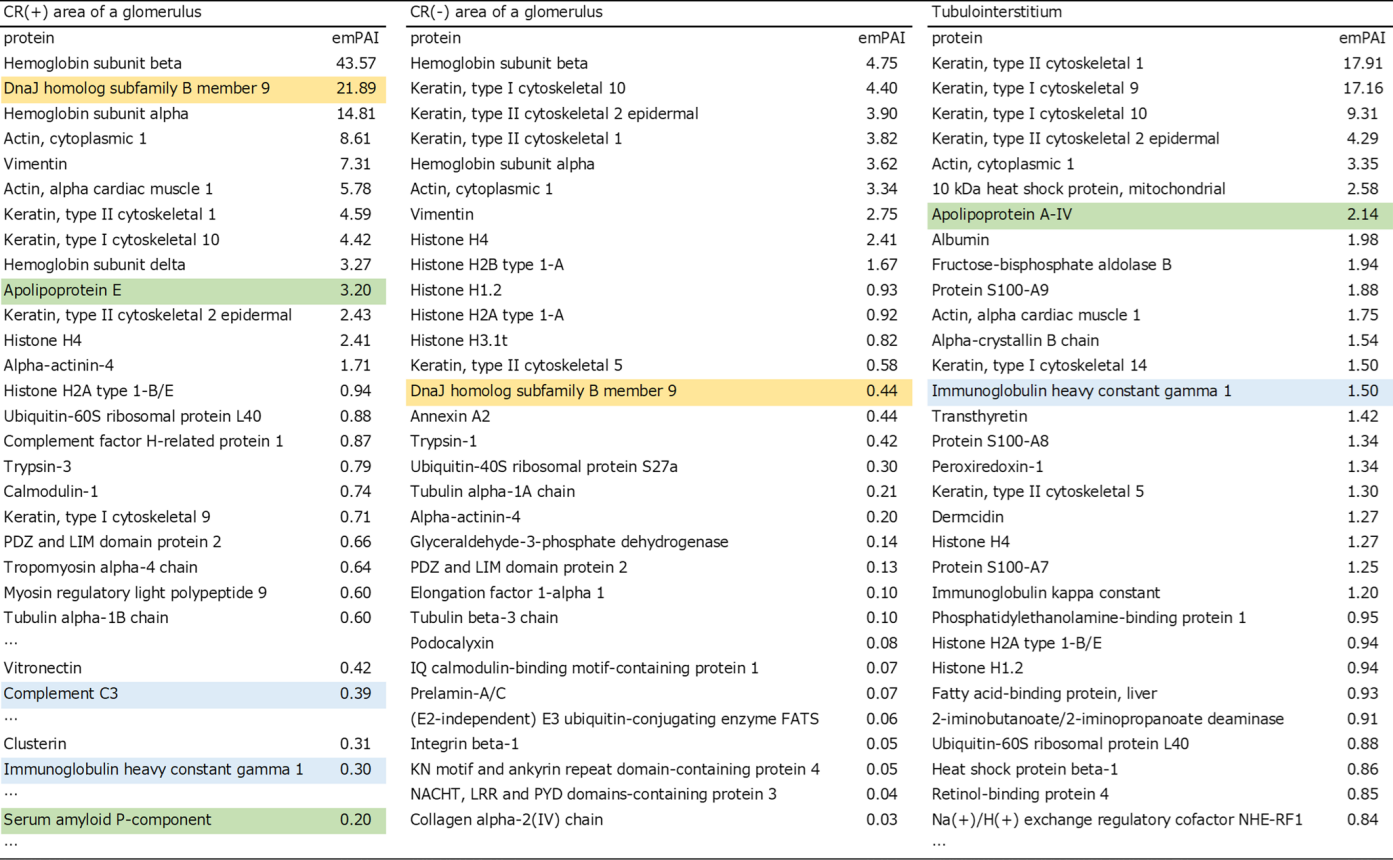
List of proteins of the transplanted kidney detected by LC-MS/MS. The yellow, green, and blue markers indicate DNAJB9, amyloid signature proteins, and immunoglobulin heavy gamma 1 and C3, respectively
  **Fig. 6:**
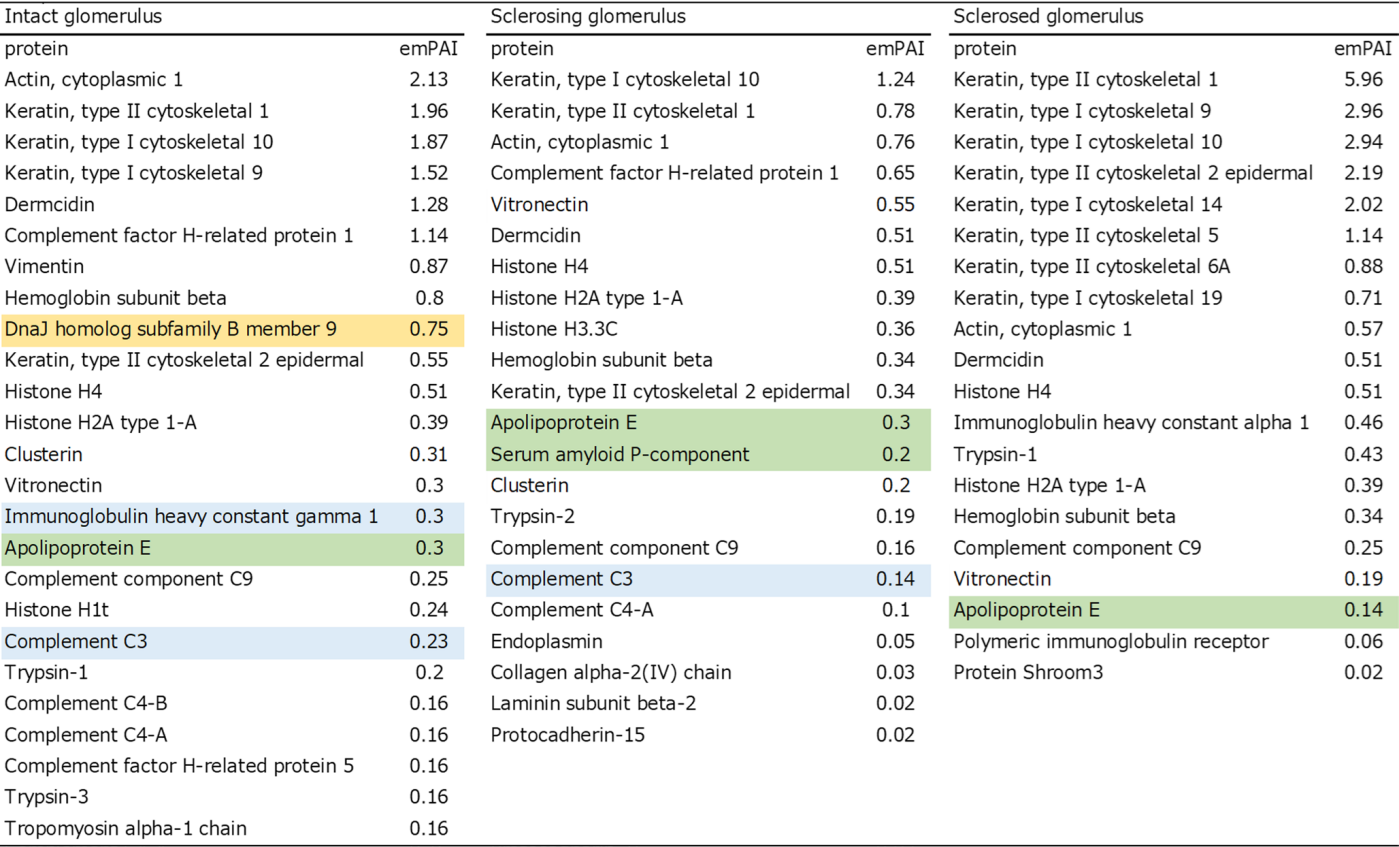
List of proteins of the native kidney detected by LC-MS/MS. The yellow, green, and blue markers indicate DNAJB9, amyloid signature proteins, and immunoglobulin heavy gamma 1 and C3, respectively
3. Additional Examinations
  Amyloid signature proteins (ASPs) such as APOE, APOA4, and serum amyloid P (SAP) were identified in the transplanted kidney’s CR-positive glomeruli and tubulointerstitium (Fig. 5). Some ASPs were also detected in the native kidney (Fig. 6).
  IF for SAP was performed and was positive, mainly in the mesangial area (Fig. 4C).
  We performed immunohistochemistry for DNAJB9 on the transplanted kidney as previously described [8] with anti-DNAJB9 antibody (HPA041553, Merck KGaA, Darmstadt, Germany), confirming the positivity in the mesangial regions (Fig. 4D). In the native kidney, IF for SAP and immunohistochemistry for DNAJB9 could not be performed due to the limited sample size, which had been fully utilized for LC-MS/MS.

## Discussion

FGN has traditionally been diagnosed based on EM findings of amyloid-like microfibrillar deposits, which are CR–negative under polarized light microscopy. However, the identification of DNAJB9 as a sensitive and specific biomarker for FGN has reshaped the diagnostic criteria. Since then, rare cases of CR–positive FGN with DNAJB9 positivity have been reported, thereby broadening the recognized spectrum of the disease.

Two English-language case series and two case reports on CR-positive FGN were found on PUBMED [7, 11–13] (Table 1). Congophilic FGN is rare; the reported frequency is 4–5% of FGN cases. The patients’ ages ranged from 29 to 82. The female-to-male ratio was 16:14. Major background diseases were hypertension, diabetes, and HCV infection. Common histological features were mesangioproliferative and MPGN patterns. IF showed IgG positivity in all cases and C3 in many cases. Fibril distribution on EM was mesangium and GBM in almost all cases. Fibril width ranged from 9 to 20 nm. To summarize the previous reports on congophilic FNG, there is no significant difference in age, sex, background disease, clinical symptoms, pattern of injury, and IF between CR positive FGN and CR negative FGN. The only notable difference is the fibril diameter on EM. It is intriguing that in the CR-positive areas of the present case, microscopic spiculae from the GBM, a characteristic finding of renal amyloidosis, were evident on the PAM stain. The 9–15 nm-wide fibrils identified on EM were slightly wider than typical amyloid fibrils (8–10 nm) and thinner than the typical FGN fibrils (12–24 nm). This atypical width of fibrils suggests a possible association between congophilic properties and thinner fibrils in CR positive FGN.

**Table 1:** Clinical and pathological characteristics of congophilic FGN and general FGN

	FGN (Congophilic)					FGN (general)	
	Alexander [11]	Andeen [7]	Goto [12]	Gandhi [13]	Our case	Nasr [3]	Rosenstock [16]
No. of patients	18	9	1	1	1	66	61
Age	55–82	n/a	29	58	60	19–81 (mean, 53)	28–81 (mean, 56.8)
Sex, F/M	11/7	5/4	M	M	M	30/36	24/37
Associated medical condition	HTN, 9 (50%); DM, 7 (39%); HCV, 4 (22%); gout, 2 (11%); obesity, 2 (11%); cancer, 3 (17%); NSAID use, 2 (11%); Crohn disease, 1 (6%); colonary artery disease, 2 (11%); HIV, 1 (6%); COPD, 1 (6%); nephrolithiasis, 1 (6%)	RA, 1; untreated HCV, 1; intravenous drug use, 1; hypocomplementemia, 1; EGPA, 1	n/a	treated HCV	HTN	Malignancy, 15 (23%); DM, 13 (20%); autoimmune, 10 (15%); coronary artery disease, 6 (9%); COPD, 3 (5%); HCV, 2 (3%)	Hypertension, 47 (77%); DM, 12 (20%); lymphoproliferative disease, 1 (2%); carcinomas, 3 (5%); SLE, 1 (1.6%); autoimmune/vasculitis, 2 (3%); cirrhosis/hepatitis, 3 (5%); HCV, 6/34 (17%)
Serum creatinine, mg/dL	1.1–7 (median, 1.6)	n/a	n/a	3.2	0.81	0.5–8.3 (mean, 2.1)	0.5–14 (mean 3.1)
Proteinuia, g/day	0.4–20	n/a	yes	yes	yes	0.2–20.4 (mean, 5.62)	0.84–25 (mean, 6.4)
Nephrotic syndrome	8 (44%)	n/a	n/a	no	yes	24/64 (38%)	28/53 (52%)
Serum M protein	6 (33%)	1(11%)	n/a	n/a	no	6 (9%)	6/45 (13%)
ESRD	5 (28%)	n/a	n/a	no	yes (native kidney)	27/61 (44%)	25/56 (45%)
Sclerosed glomeruli, %	3–73%	n/a	n/a	n/a	28% (native kidney); 7% (transplanted kidney)	mean, 25%	n/a
Pattern of injury	MES, 10 (56%); MPGN, 4 (22%); mesangial expansion, 2 (11%); nodular glomerulosclerosis 1 (6%)	Crescent, 3 (33%)	MES	MES	MES (native kidney); mesangial expansion (transplanted kidney)	MES, 47 (71%); MPGN, 10 (15%); endocapillary proliferative, 4 (6%); crescentic, 3 (5%)	MPGN, 27 (44%); MES, 13 (21%); DPGN, 9 (15%); MGN, 4 (7%); DS, 8 (13%)
IF	IgG, 18(100%); C3, 13 (72%); IgM, 9 (50%); IgA, 5 (28%); C1q, 5 (28%)	IgG, 9 (100%)	IgG( +)	IgG( +), C3( +)	IgG( +), C3( +)	IgG, 66/66 (100%); IgM, 30/65 (47%); IgA, 18/65 (28%); C3, 59/64 (92%); C1q, 38/63 (60%); kappa, 52/61 (85%); lambda, 55/61 (90%)	IgG, 96%; IgM, 52%; IgA, 30%; C3, 83%; C1q, 41%
Fibril distribution on EM	Mesangial and GBM, 17 (94%); Mesangial, 1 (6%)	n/a	n/a	Mesangial and GBM	Mesangial and GBM	Mesangial, 65 (98%); GBM, 56 (85%)	Mesangial, 98%; GBM, 92%
Fibril diameter, nm	11–18 (median, 13)	median, 15	10–20	14.8	10–15 (netive kidney); 9–15 (transplanted kidney)	9–26 (mean, 18.1)	13–29 (mean, 20.1)
MS	DNAJB9 and ASP (APOA4, APOE, SAP)	n/a	DNAJB9, ASP (APOE, vitronectin, SAP)	n/a	DNAJB9, ASP (APOE, APOA4, SAP)	n/a	n/a

n/a, not available; HTN, hypertention; DM, diabetes mellitus; HCM, hepatitis C virus; NSAID, non-steroidal anti-inflammatory drug; HIV, humanimmunodeficiency virus; COPD, chronic obstructive disease; RA, rheumatoid arthritis; EGPA, eosinophilic granulomatosis with polyangiitis; SLE, systemic lupus erythematosus; MPGN, membranoproliferative glomerulonephritis; MES, mesangioproliferative; DPGN, diffuse proliferative glomerulinephritis; MGN, embranous glomerulonephrits; DS, diffuse sclerosing

Advancements in LC-MS/MS-based proteomic analysis have provided insights into the pathophysiology of amyloidosis and a new protein classification system related to amyloidosis. ASPs consisting of APOE, SAP, and APOA4 have been reported to be excellent surrogates for diagnosing amyloidosis when at least two were detected by LC-MS/MS [14]. Alexander et al. studied 18 cases of congophilic FGN and also reported a small amount of ASP by MS in addition to DNAJB9 in congophilic FGN cases; however, they concluded that congophilic FGN is proteomically “identical” to traditionally defined FGN [11]. Alexander et al. suggested that congophilia is due to the increased amount of APOE deposit. In the present case, we performed precise LMD to analyze the areas of interest in the glomerulus separately by LC-MS/MS. As a result, DNAJB9 and two ASPs, SAP and APOE, were detected in the CR-positive areas in the recurrent FGN. The quantity of these ASPs was relatively small, and the possibility of detecting these proteins in serum cannot be excluded. However, we also performed IF for SAP, which showed positive staining in mesangial areas, and DNAJB9 positivity was also observed in immunohistochemical staining (Fig. 4C-D). The detection of DNAJB9 alongside SAP in CR-positive areas suggests a potential co-deposition mechanism. The identification of microfibrils in the mesangium on EM substantiates this suggestion. Further study is necessary to elucidate the mechanism of congophilia in a small subset of FGN.

There are few reports of FGN recurrence after transplantation; two case series are presented. El Ters et al. followed 14 cases of FGN for a median of 5.7 years; one case had confirmed FGN recurrence by biopsy five years after transplantation, and 2 cases had recurrence after ten years [6]. Andeen et al. reported that at a median follow-up time of 7 years after renal transplantation, 1 of 8 patients (12.5%) was confirmed to have recurrent FGN 3 months after transplantation [7]. In previous reports, the time to recurrence varied widely from 3 months to 10 years. The recurrence of FGN in our case occurred at an intermediate time point of two years and eight months post-transplant, aligning with previously reported recurrence timelines. On the other hand, there have been no reports of recurrent congophilic FGN; this is the first report of such a recurrence.

FGN is usually a rapidly progressive disease. The FGN case with the longest clinical course in the literature was reported by Micarelli et al., where the patient developed ESRD 26 years after diagnosis of FGN [15]. They concluded that the factors contributing to the indolent clinical course included the favorable histologic subtype, clinical presentation with good renal function, and treatment with renin-angiotensin system inhibitors. Rosenstock et al. reported that among various histological types of FGN, mesangial proliferative/sclerosing and membranous glomerulonephritis patterns are associated with the most favorable prognosis, with a time to ESRD of over 80 months [16]. Other reported predictors of progression to ESRD include older age, higher creatinine and proteinuria at biopsy, the severity of interstitial fibrosis, and a higher percentage of global glomerulosclerosis [3, 16]. In the present case, the clinical course was even longer; the patient took approximately 40 years to develop ESRD from the onset of proteinuria. A renal biopsy was performed for the first time over 30 years after the onset of proteinuria, showing a mesangial proliferative pattern. The younger age at the onset of proteinuria (around 20 years), the low-grade proteinuria, and the favorable histological type most likely contributed to this indolent clinical course.

On the other hand, after transplantation, nephrosis occurred in about two years, and recurrence of FGN was confirmed with the biopsy material taken at 2 years and 8 months after the transplantation. There are very few reports describing the clinical course before and after transplantation. Mitwalli, et al. [17] described a case with a clinical course of at least one year before transplant and recurrence five months after transplant. To the best of our knowledge, there are no other studies comparing the length of the clinical course before and after the transplantation for FGN.

The markedly faster development of recurrent FGN in the transplanted kidney compared with the native kidney in the present case may indicate the involvement of a systemic condition underlying microfibril deposition and DNAJB9, which remains unaffected by renal transplantation. This observation underscores the need to investigate circulating factors or host-related conditions that could contribute to disease recurrence after transplantation.

We presented a case of congophilic FGN recurring in an allograft kidney two years and eight months post-transplant. The primary disease demonstrated an unusually long, indolent clinical course. The discovery of DNAJB9 significantly contributed to defining this entity; however, its clinical and pathological presentations can vary. It is important to be aware that FGN can manifest characteristic findings of amyloidosis microscopically and, in some cases, on EM. In such cases, LC-MS/MS or immunohistochemistry for DNAJB9 is essential to reach a correct diagnosis and ensure proper patient management.
