# Correction: Congophilic fibrillary glomerulonephritis recurrence post-renal transplant: diagnostic challenges and proteomic insights

**DOI:** 10.1007/s13730-026-01137-y

**Published:** 2026-06-29

**Authors:** Hiroshi Watanabe, Michiko Nagamine, Yukako Shintani-domoto, Kunio Kawanishi, Kousuke Ishino, Tsukasa Nakamura, Shinji Sumiyoshi, Eiichi Konishi

**Affiliations:** 1 Department of Surgical Pathology, Kyoto Prefectural University of Medicine, Kyoto, Japan; 2 Department of Diagnostic Pathology, Nippon Medical School Hospital, Tokyo, Japan; 3 Department of Anatomy, Showa Medical University School of Medicine, Tokyo, Japan; 4 Department of Integrated Diagnostic Pathology, Nippon Medical School, Tokyo, Japan; 5 Division of Transplant Surgery, Department of Surgery, Massachusetts General Hospital, Boston, USA; 6 Department of Organ Transplantation and General Surgery, Kyoto Prefectural University of Medicine, Kyoto, Japan; 7 Department of Diagnostic Pathology, Tenri Hospital, Nara, Japan

**Correction to:** 10.1007/s13730-025-01064-4

In this article, Fig(s) 1, 2, 3, 4, 5 and 6 ordered incorrectly; the figure(s) should have appeared as shown below.

**Fig. 1:**
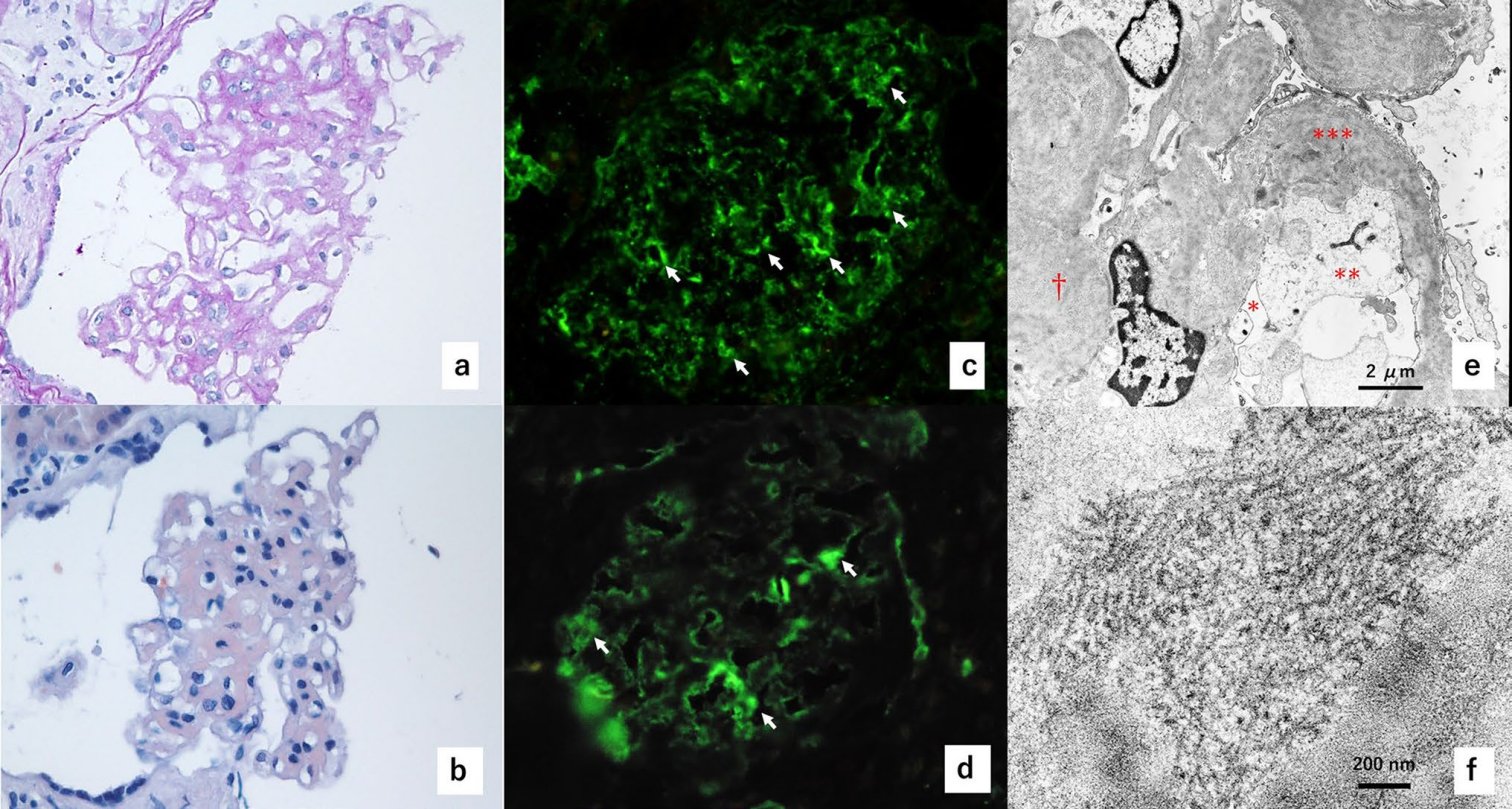
Findings on biopsy of native kidney. Original magnification, ×400 for A) and B). **A** The native kidney showed mesangial proliferation on the PAS stain. **B** The glomerular tuft showed thickening, but there was no green birefringence under polarized light on the Congo red stain. The IF was positive for **C** IgG and **D** C3. **E** EM showed severe GBM thickening and microfibril deposition in the GBM and the mesangial area. *, fenestrae; **, endothelial cells; ***, GBM; †, mesangium (×3000). **F** The diameter of the fibrils was 10–15 nm (×20000)

**Fig. 2:**
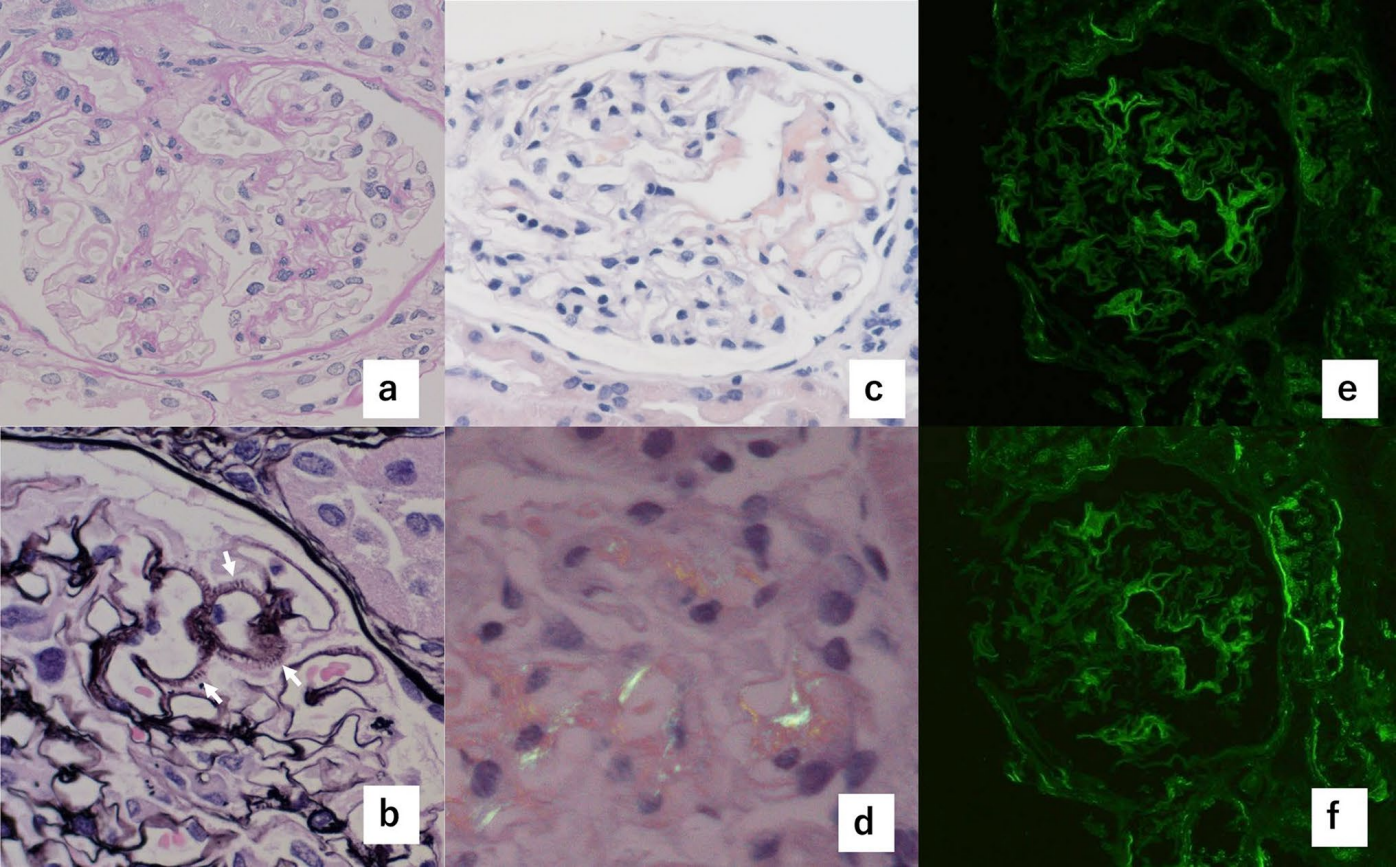
Findings on biopsy of transplanted kidney, taken two years and eight months post-transplant. Original magnification, ×400 for A) through D). **A** No aberrant proliferation of mesangial cells is seen on the PAS stain. **B** PAM stain shows focal/segmental spiculae extending outwards from the GBM. **C** Congo red was positive on mesangium and glomerular tuft. **D** Congo red stain shows green birefringence under polarized light. Immunofluorescence was positive around the vascular pole and mesangium for **E** IgG and **F** C3

**Fig. 3:**
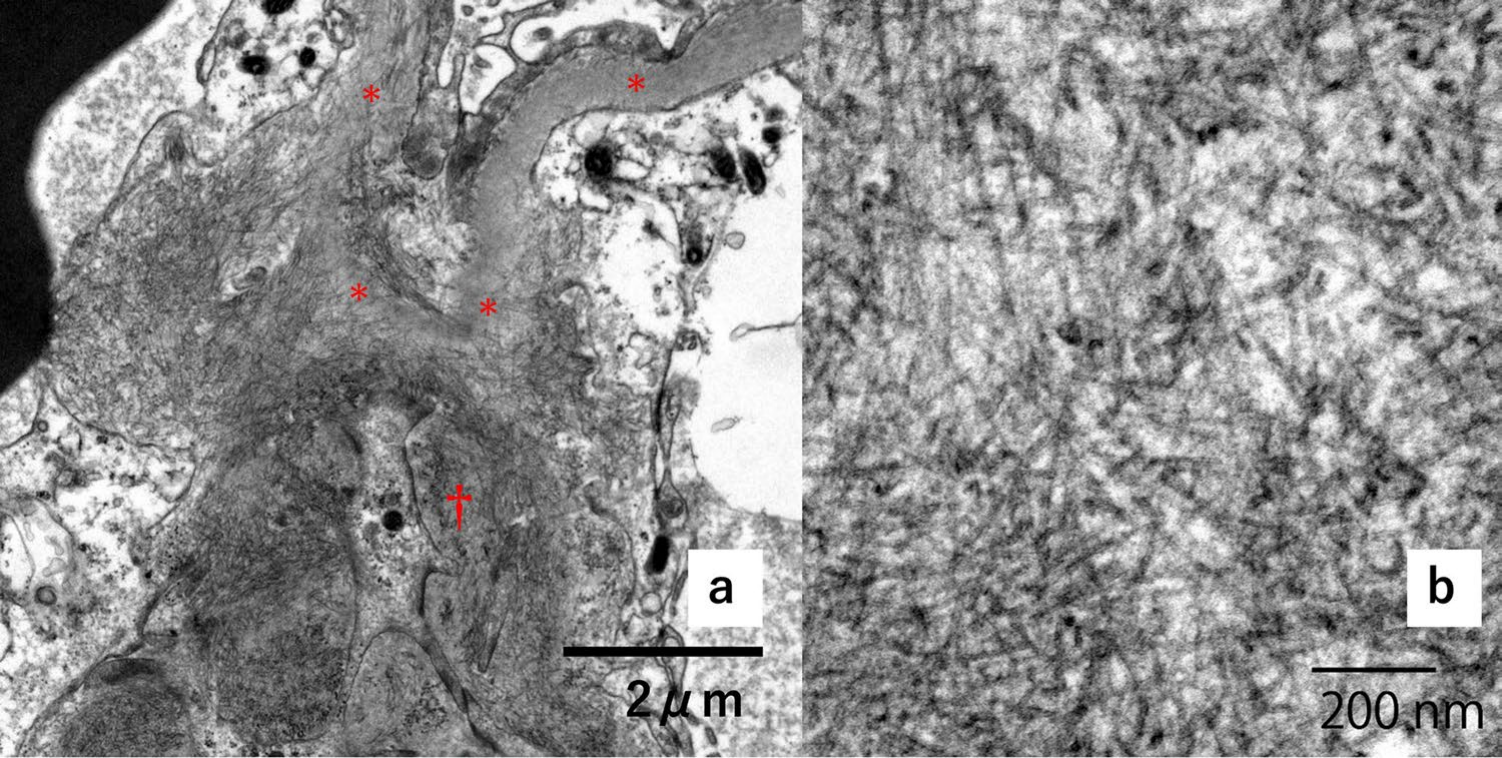
Electron microscopy revealed (**A**) fibrillar structures in the segmental subepithelial, subendothelial, and mesangial region (×8000). *, glomerular basement membrane; †, mesangium. **B** The diameter of the fibrils was 9–15 nm with a random arrangement and no branching (×50000)

**Fig. 4:**
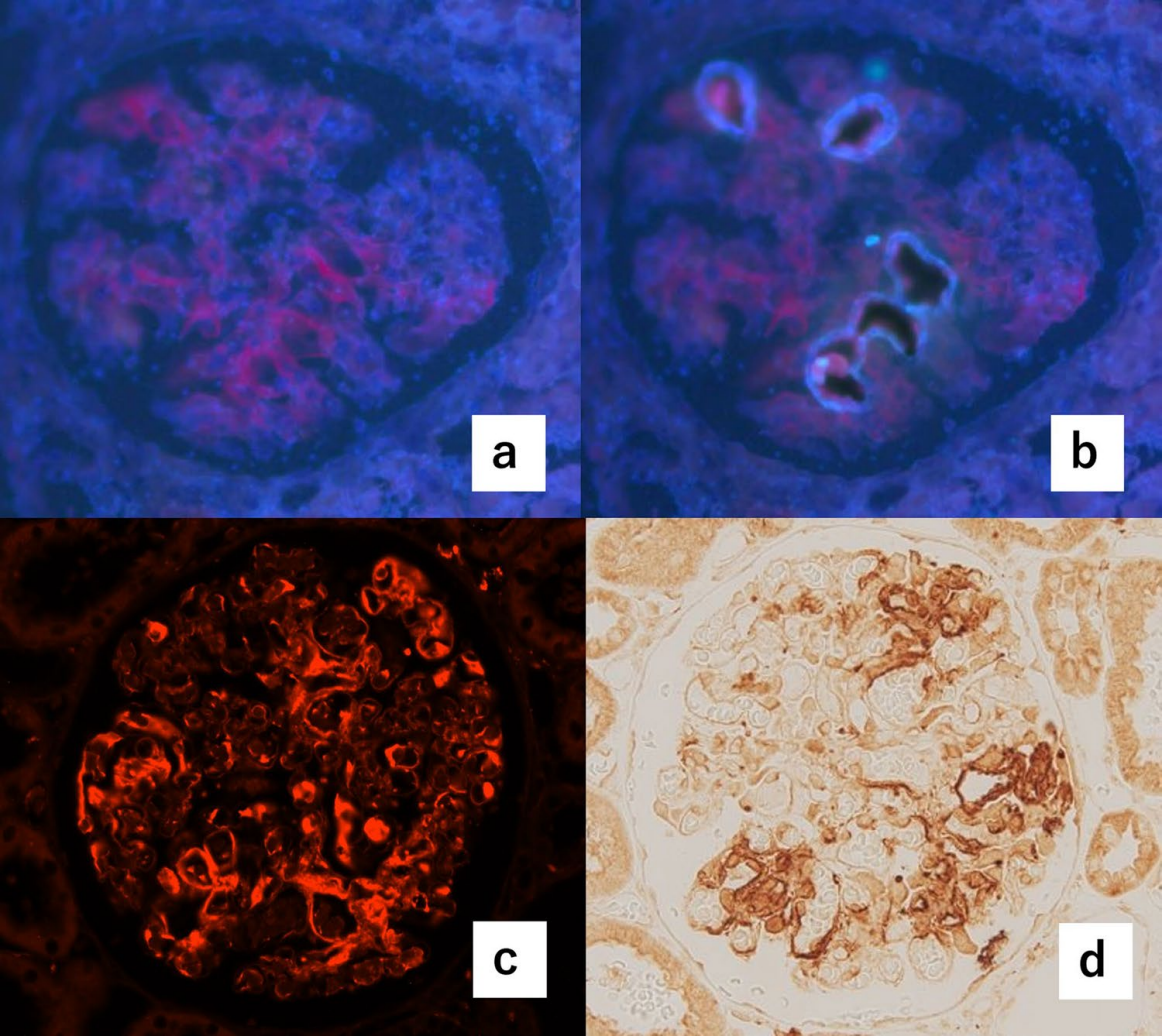
Transplanted kidney. Sampling area for MS (**A**, **B**). Immunofluorescence for SAP (**C**) and immunohistochemistry for DNAJB9 (**D**). A, B) Only areas showing Congo red positivity were collected by laser microdissection for mass spectrometry. C) SAP immunofluorescence shows positivity in the mesangial region. D) DNAJB9 immunohistochemical stain shows positivity in the mesangial region

**Fig. 5:**
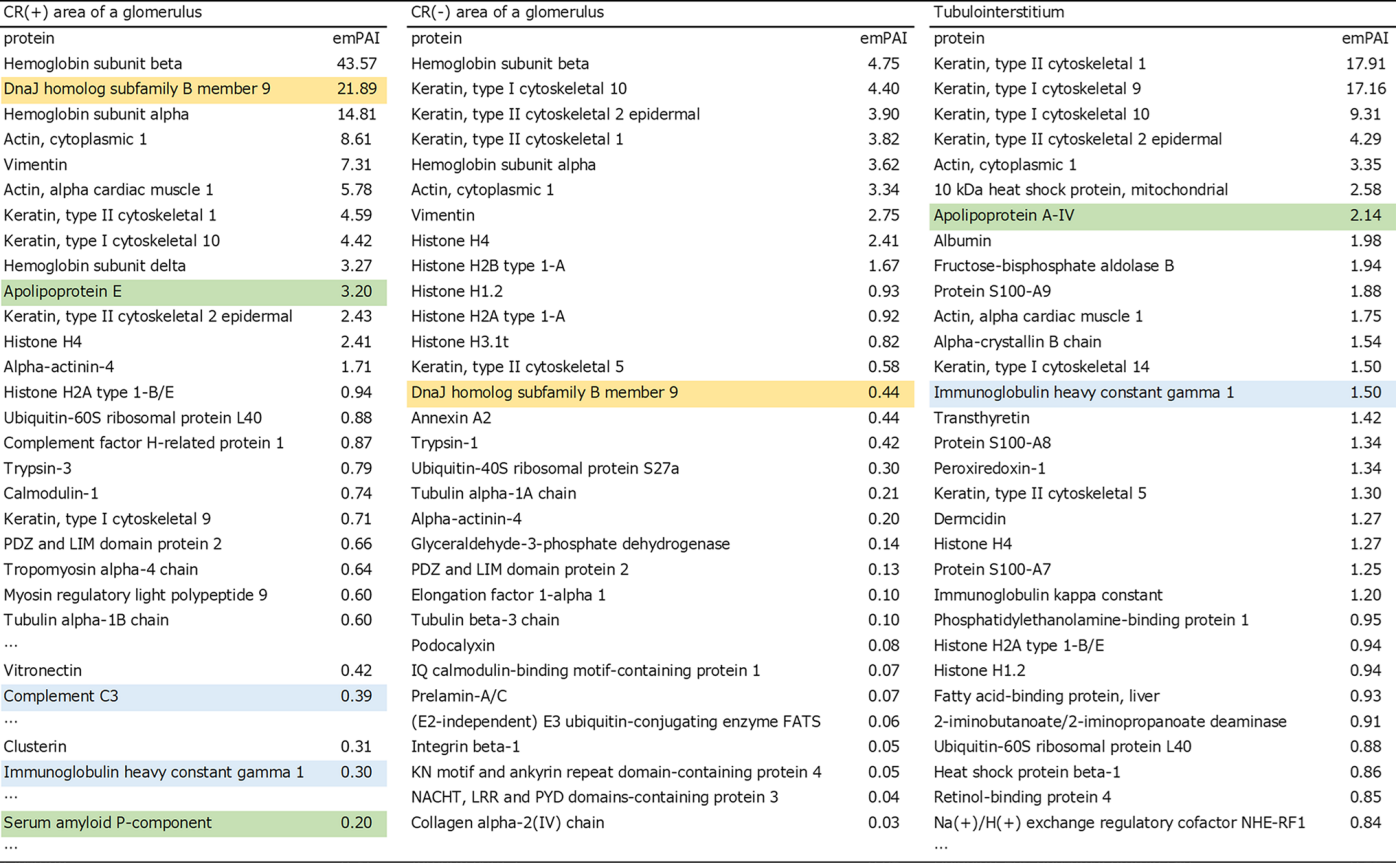
List of proteins of the transplanted kidney detected by LC-MS/MS. The yellow, green, and blue markers indicate DNAJB9, amyloid signature proteins, and immunoglobulin heavy gamma 1 and C3, respectively

**Fig. 6:**
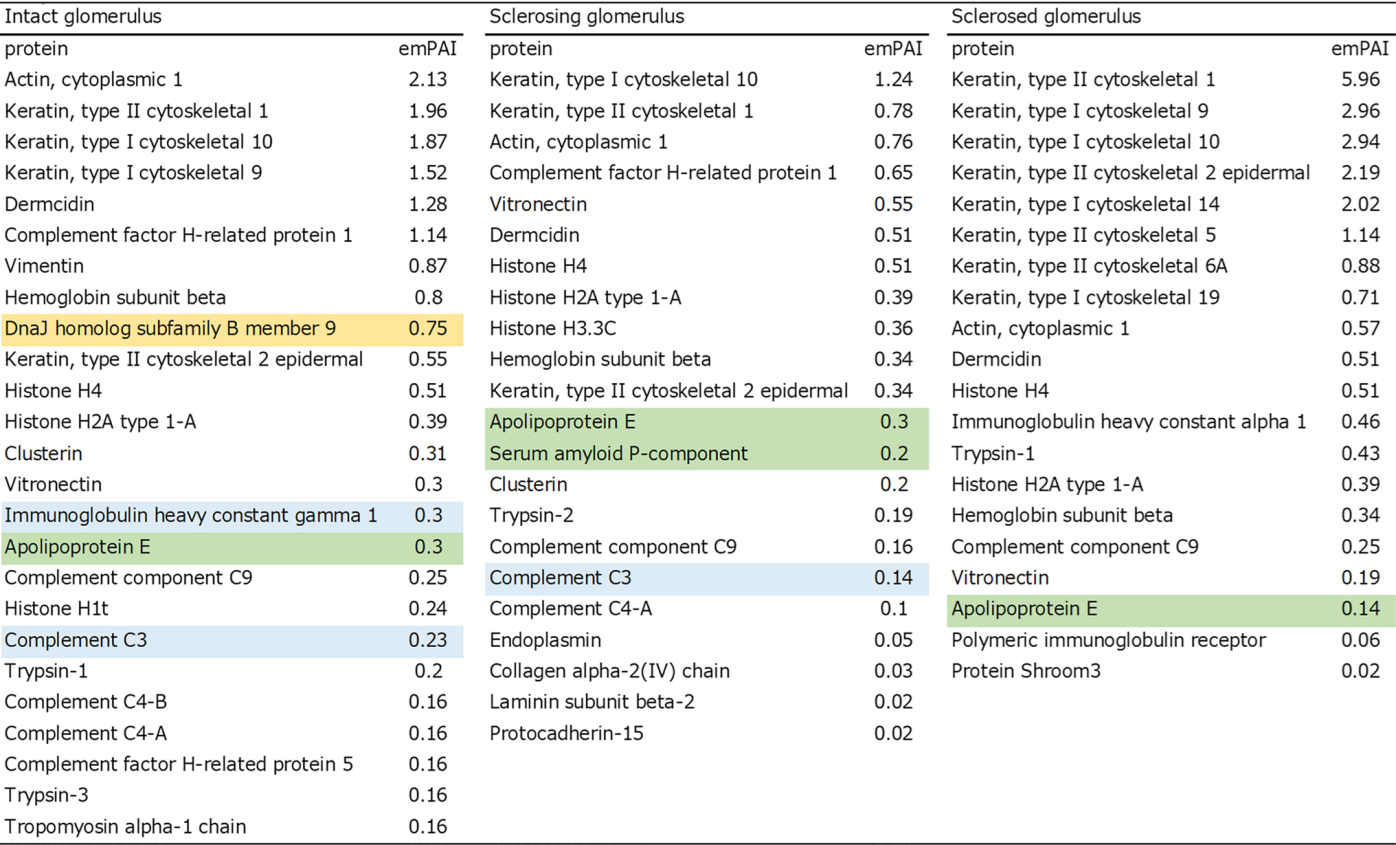
List of proteins of the native kidney detected by LC-MS/MS. The yellow, green, and blue markers indicate DNAJB9, amyloid signature proteins, and immunoglobulin heavy gamma 1 and C3, respectively

The original article has been corrected.

